# Hyperoxia During Exercise: Impact on Adenosine Plasma Levels and Hemodynamic Data

**DOI:** 10.3389/fphys.2020.00097

**Published:** 2020-02-11

**Authors:** Alain Boussuges, Sarah Rives, Marion Marlinge, Guillaume Chaumet, Nicolas Vallée, Régis Guieu, Olivier Gavarry

**Affiliations:** ^1^ERRSO, Institut de Recherche Biomédicale des Armées (IRBA), Toulon, France; ^2^Center for Cardiovascular and Nutrition Research (C2VN), Aix-Marseille Université, INSERM, INRA, Marseille, France; ^3^AltraBio, Lyon, France; ^4^Laboratoire Impact de l’Activité Physique sur la Santé, UFR STAPS, Université de Toulon, La Garde, France

**Keywords:** oxygen, circulatory system, hyperemia, echocardiography, cardiac output, systemic vascular resistances

## Abstract

**Introduction:**

Adenosine is an ATP derivative that is strongly implicated in the cardiovascular adaptive response to exercise. In this study, we hypothesized that during exercise the hyperemia, commonly observed during exercise in air, was counteracted by the downregulation of the adenosinergic pathway during hyperoxic exposure.

**Methods:**

Ten healthy volunteers performed two randomized sessions including gas exposure (Medical air or Oxygen) at rest and during exercise performed at 40% of maximal intensity, according to the individual fitness of the volunteers. Investigations included the measurement of adenosine plasma level (APL) and the recording of hemodynamic data [i.e., cardiac output (CO) and systemic vascular resistances (SVR) using pulsed Doppler and echocardiography].

**Results:**

Hyperoxia significantly decreased APL (from 0.58 ± 0.06 to 0.21 ± 0.05 μmol L^–1^, *p* < 0.001) heart rate and CO and increased SVR in healthy volunteers at rest. During exercise, an increase in APL was recorded in the two sessions when compared with measurements at rest (+0.4 ± 0.4 vs. +0.3 ± 0.2 μmol L^–1^ for medical air and oxygen exposures, respectively). APL was lower during the exercise performed under hyperoxia when compared with medical air exposure (0.5 ± 0.06 vs. 1.03 ± 0.2 μmol L^–1^, respectively *p* < 0.001). This result could contribute to the hemodynamic differences between the two conditions, such as the increase in SVR and the decrease in both heart rate and CO when exercises were performed during oxygen exposure as compared to medical air.

**Conclusion:**

Hyperoxia decreased APLs in healthy volunteers at rest but did not eliminate the increase in APL and the decrease in SVR during low intensity exercise.

## Introduction

Subjects experienced physical effort under an ambient hyperoxic environment during various professional or recreational activities. In ambient hyperbaric environment, such as the environment experienced by divers or by professional workers in a tunnel boring machine hyperbaric chamber, hyperoxia is secondary to the increase in ambient pressure and its consequence on oxygen partial pressure. In hypoxic environments, enriched oxygen mixtures are used and can lead to an increase in oxygen partial pressure. Lastly, some athletes used hyperoxic exposure during their exercise training to improve physical performance.

In healthy volunteers at rest, the impact of hyperoxia on the cardio-vascular system has been studied. A decrease in cardiac output (CO) secondary to a slowing of the heart rate has been observed (Waring et al., 2003; Thomson et al., 2006; Gole et al., 2011). Furthermore, an increase in systemic vascular resistances (SVR) and a decrease in arterial compliance induced by an arterial vasoconstriction have been commonly reported (Milone et al., 1999; Rossi and Boussuges, 2005; Gole et al., 2011).

Various mechanisms have been implicated in the alteration of vasomotion in an ambient hyperoxic environment. An increase in the endothelin I plasma level might contribute to the vasoconstriction of the cerebral arteries under hyperoxia (Armstead, 1999). Alterations to endothelial function including action of nitric oxide (NO) being impeded by reactive oxygen species have been reported (Rubanyi and Vanhoutte, 1986; Pasgaard et al., 2007). A decrease in muscle sympathetic nerve activity has been reported at rest but not during exercise (Seals et al., 1991). Recently, a decrease in adenosine plasma levels (APLs) has been reported in rats subjected to normobaric or hyperbaric hyperoxia (Bruzzese et al., 2015). Adenosine is an ATP derivative which strongly impacts heart rate and blood pressure through its G-protein coupled receptors known as A1, A2A, A2B, or A3R, depending on their pharmacological properties (Burnstock, 2017).

During exercise on land, hemodynamic changes include an increase in CO secondary to the increase in both heart rate and stroke volume and a decrease in SVR. Exercise-induced hyperemia has been attributed to various factors such as NO, prostacyclin (PGI2), and endothelium-derived hyperpolarizing factor (Sarelius and Pohl, 2010). Furthermore, it has been reported that adenosine was responsible for a part of the maintained phase of the muscle vasodilation that accompanies muscle hyperemia during exercise (Marshall, 2007).

In this study, we hypothesized that during exercise in hyperoxia, the hyperemia commonly observed during exercise in air was counteracted by the downregulation of the adenosinergic pathway secondary to the increase in oxygen partial pressure.

## Materials and Methods

### Subjects

Ten healthy male volunteers participated in this study. Mean age, weight, height, and body surface area were 35 ± 6 years, 73 ± 12 kg, 177 ± 7 cm, and 1.9 ± 0.2 m^2^, respectively. All volunteers gave their written informed consent to participation in the experiment, which was approved by the Regional Ethics Committee (Aix Marseille University, CPP-1, ID RCB: 2008-AOO171-54). The research was conducted according to the Helsinki Declaration. All the exercise bouts were performed on the same ergobike (Tunturi Endurance E80R Recumbent Bike). This recumbent bike was used to make the ultrasonographic examinations easier. Prior the main experiment, the volunteers performed an incremental maximal cycling exercise to assess maximal aerobic fitness (peak VO_2_) and the power corresponding to 40% intensity exercise on this ergometer.

### Main Experiment

The experimental session consisted of a sequence of four measurement periods: baseline, gas exposure (medical air or oxygen), cycling exercise at a workload corresponding to 40% of VO_2_ peak (breathing air or oxygen), and recovery period, 30 min after the end of exercise.

To determine data at rest, participants were in a sitting position, in a quiet and air-conditioned room (50% humidity, temperature: 25°C). During exercises, ambient temperature was lowered to 22°C.

During the baseline period, volunteers breathed ambient air without a face mask for 10 min of quiet rest. After the baseline investigations, each subject underwent two sessions in a randomized order. The volunteers at rest were exposed during 1 h to a medical gases supplied by the society Air Liquide (AL Healthcare, Paris, France), i.e., medical air (21% oxygen and 79% nitrogen) or pure oxygen (inspired oxygen fraction = 1) delivered in a Douglas bag, to be breathed at atmospheric pressure. Subjects received either medical air or pure oxygen through a face mask connected to two-way low-resistance *T* valve (Hans Rudolph Inc., Shawnee, KS, United States). The facemask was fixed firmly around the mouth and nose to prevent air leaks. An oxygen analyzer (Servomex Oxygen Analyzer 570A; Servomex Group Ltd, Crowborough, United Kingdom) was used to control FiO_2_.

Volunteers and investigators were blinded to the gas mixture used. Thereafter, all participants performed a 30-min constant-load exercise on the recumbent bike, breathing medical air or oxygen. The pedaling rate was fixed at 60 rotations per minute. The workload was adjusted to represent 40% of the VO_2_ peak according to the individual maximal incremental exercise. There were a minimum of 3 days and a maximum of 7 days between the two sessions. The two sessions were performed in a random order, at the same time of day for each subject. At each measurement period, the investigations included venous blood sampling, and an ultrasonographic study.

### Investigations

All procedures were undertaken after 10 min of rest as baseline measurements, 1 h after the exposure to medical air or oxygen at rest, 20 min after the beginning of the exercises, and during recovery, 30 min after the return to ambient air. During the experiment, oxygen saturation level (SpO_2_) was measured using a pulse oximeter (NPB 40; Nellcor Puritan Bennett, Pleasanton, CA, United States) fixed on the left median finger.

#### Echocardiographic and Doppler Study

The ultrasonographic examinations were carried out by an experienced investigator using a Doppler cardiovascular ultrasound (Mylab 25CV, Esaote SpA, Genova, Italy) connected to a 2.5–3.5 MHz transducer array. The mean duration for each examination was 10 min. All Doppler recordings were performed at the end of a normal expiration in order to eliminate the effects of breathing on the parameters studied. Measurements were averaged from at least three different beats.

The left ventricular outflow tract (LVOT) was first measured by 2D echocardiography from the left parasternal long axis view. The aortic systolic flow velocity time integral (AoVTI) was measured by computer-assisted determination from the pulsed-wave Doppler profile of the aortic blood flow from the apical four chamber view, allowing stroke volume (SV) and CO to be calculated: SV = AoVTI × LVOT, CO = SV × HR. SVR were calculated as mean arterial pressure (MAP) divided by CO.

#### Blood Pressure Measurement

Sphygmomanometer blood pressure measurements on the right arm were obtained at the end of each echographic examination. MAP was calculated as DAP + 1/3 (SAP – DAP), where SAP and DAP were respectively the systolic and diastolic arterial blood pressures.

#### Adenosine Plasma Level Measurement

Blood sample collection for adenosine plasma measurement was performed through a catheter inserted in a vein in the elbow folds, at baseline, during gas exposure at rest and during exercise (at the end of each period) and after recovery as described above. Since adenosine has a short half-life, we used a stop solution to block adenosine metabolism and uptake by red blood cells as previously described (Guieu et al., 1994; Maille et al., 2019). After centrifugation (1500 × *g*, 10 min, 4°C), supernatants were pipetted off and frozen (−80°C) until analysis. APL measurement was performed by LC-MS/MS as previously described (Maille et al., 2019).

### Statistical Analysis

Data are expressed as mean ± SEM. Statistical tests were run on Sigma Stat software.

The cohorts for comparison consisted of the ten subjects at 4 time points during the two sessions, i.e., under medical air or oxygen exposure: baseline, during gas exposure at rest, during exercise and 30 min after the end of exercise. Comparison between cohorts of continuous variables having normal distribution was carried out with the parametric analysis of variance (repeated measurements, ANOVA); multiple pairwise comparison procedures were done using the Holm-Sidak method. In the case of variable cohorts not having normal distribution, comparisons were performed with a non-parametric univariate analysis (Friedman’s test); comparison of dichotomous variables was carried out using the Student-Newman–Keuls Method. Differences between groups were considered significant at *p* < 0.05.

## Results

### Exercise Testing

The peak of VO_2_ and maximal workload recorded by the incremental exercise testing were 47 ± 6 mL kg^–1^ min^–1^ (115 ± 15% of the predicted value) and 263 ± 45 Watts, respectively. According to these results, the workload used for the cycling exercise performed under medical air and oxygen exposure was 115 ± 10 W. This workload corresponded to an intensity that was under the ventilatory threshold (in mean 167 ± 32 W) in all volunteers.

### SpO_2_ Measurement

SpO_2_ varied between 99 and 100% during oxygen session (at rest and exercise). During air medical session, mean SpO_2_ was 97 ± 1%.

### Adenosine Plasma Level Measurement

**FIGURE 1:**
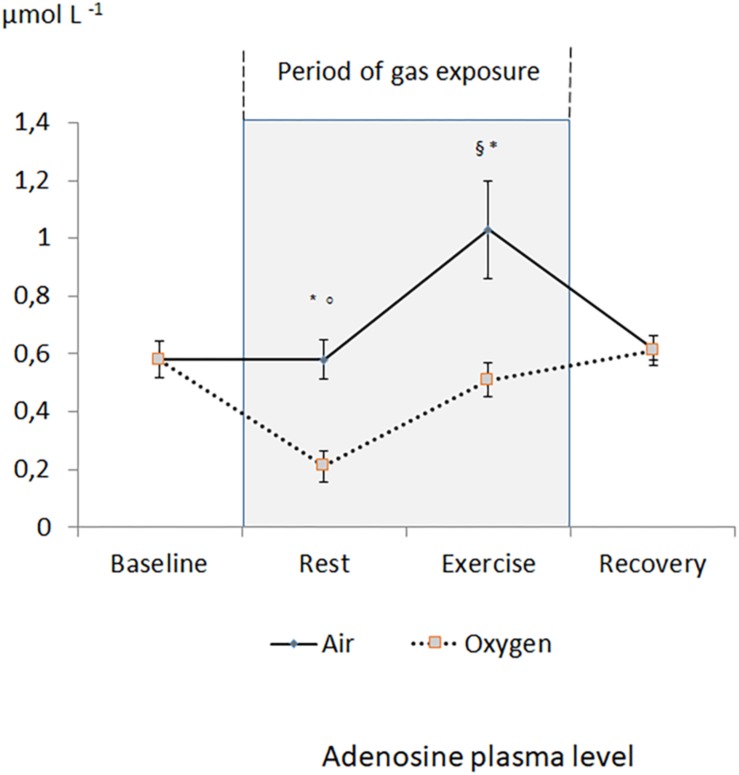
Evolution of adenosine plasma level during the two sessions. ^§^*p* < 0.05 oxygen vs. medical air. ^∗^*p* < 0.05 exercise vs. measurement at rest of the same session. °*p* < 0.05 oxygen vs. baseline.

Figure 1 reports the changes in APL during the two sessions. During the medical air session, APL significantly increased during exercise and returned to the baseline value during recovery. During oxygen exposure at rest, APL was significantly lower when compared with medical air exposure (*p* < 0.001). During exercise, APL significantly increased but remained significantly lower during oxygen exposure in comparison with medical air exposure (*p* < 0.001). After exercise, APL returned to the baseline values.

There is no significant difference between the increase in APL during exercises between the two sessions (+0.4 ± 0.4 μmol L^–1^ vs. +0.3 ± 0.2 μmol L^–1^ for medical air and oxygen exposures, respectively). Interestingly, APL at the end of exercise in O_2_ ambiance was similar than APL at rest in medical air ambiance.

### Blood Pressure Measurement

Systolic blood pressure increased during exercise, this increase was significantly larger during oxygen rather than medical air exposure (*p* < 0.05). Mean and diastolic blood pressure significantly increased during exercise without a significant difference between the two sessions (Table 1).

**TABLE 1:** Blood pressure and hemodynamic data.

Parameters	Session	Baseline (AA)	Gas exposure	Exercise	Recovery (AA)
SBP (mmHg)	Medical air	115 ± 5	107 ± 4	136 ± 6*	110 ± 3
	Oxygen	117+/2	116 ± 2	145 ± 5^*§^	107 ± 4^#^
MBP (mmHg)	Medical air	88 ± 4	82 ± 3	97 ± 4*	85 ± 3
	Oxygen	90 ± 3	85 ± 2	102 ± 4*	81 ± 4^#^
DBP (mmHg)	Medical air	75 ± 4	70 ± 3	78 ± 3*	73 ± 4
	Oxygen	76 ± 3	70 ± 3	81 ± 5*	69 ± 5^#^
PP (mmHg)	Medical air	41 ± 2	37 ± 2	59 ± 5*	38 ± 4
	Oxygen	42 ± 2	45 ± 3	65 ± 5*	38 ± 2
SV (mL)	Medical air	68 ± 3	73 ± 4	91 ± 8*	56 ± 5^£#^
	Oxygen	67 ± 4	65 ± 5	90 ± 10*	58 ± 5^#^
CO (L min^–1^)	Medical air	4.4 ± 0.3	4.6 ± 0.3	10.1 ± 0.7*	4.2 ± 0.4
	Oxygen	4.4 ± 0.3	3.6 ± 0.3^§⁣∘^	9.2 ± 0.7^*§^	4 ± 0.3

Results are reported as mean ± SEM. AA, ambient air; SBP, systolic blood pressure; MBP, mean blood pressure; DBP, diastolic blood pressure; PP, pulse pressure; SV, stroke volume; CO, cardiac output. * *p* < 0.05 exercise vs. other measurement times in the same session, ^§^*p* < 0.05 oxygen vs. medical air, ^£^*p* < 0.05 recovery vs. gas exposure, #*p* < 0.05 recovery vs. baseline,°*p* < 0.05 gas exposure vs. baseline.

### Hemodynamic Data

During medical air exposure at rest, hemodynamic data were similar to measurements performed at baseline. During exercise, an increase in CO secondary to an increase in both heart rate and stroke volume was observed. Blood pressure (systolic, mean, diastolic, and pulse pressure) increased during exercise whereas SVR decreased significantly. During recovery, heart rate decreased (*p* < 0.05) but remained significantly accelerated when compared with baseline values (Table 1).

**FIGURE 2:**
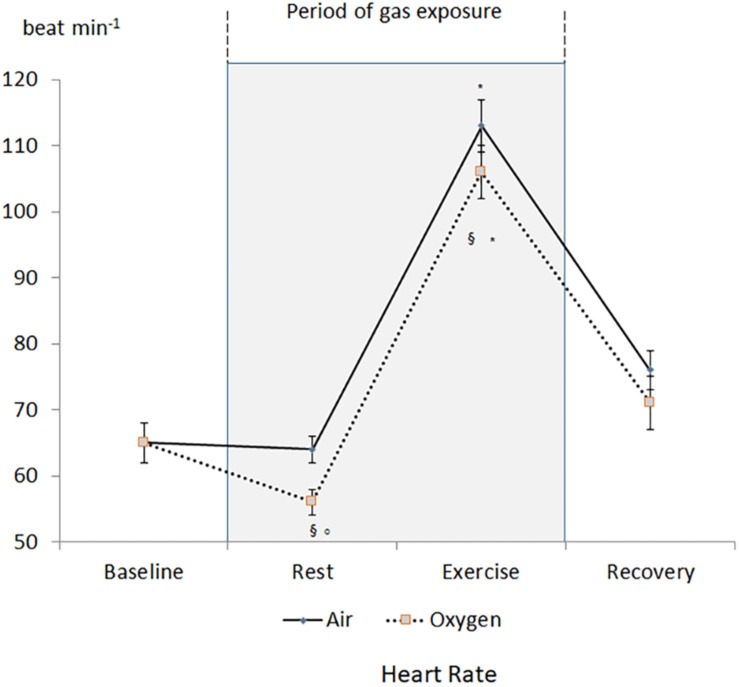
Changes in heart rate during the two sessions. ^§^*p* < 0.05 oxygen vs. medical air. ^∗^*p* < 0.05 exercise vs. measurement at rest of the same session. °*p* < 0.05 oxygen vs. baseline.

**FIGURE 3:**
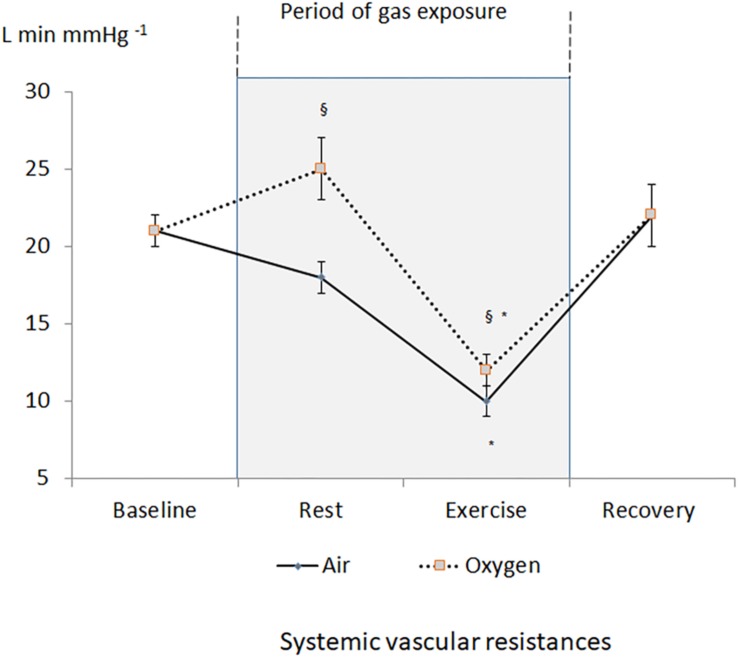
Changes in systemic vascular resistances during the two sessions. ^§^*p* < 0.05 oxygen vs. medical air. ^∗^*p* < 0.05 exercise vs. measurement at rest of the same session.

A decrease in heart rate leading to a decrease in CO was recorded during oxygen exposure at rest (Figure 2). The difference was significant with measurements performed both at baseline and during medical air exposure. SVR significantly increased during hyperoxia when compared with medical air (Figure 3).

During exercise, heart rate and CO increased but remained lower during oxygen exposure when compared with medical air, in contrast stroke volume was not significantly different between the two conditions (Table 1). During exercise SVR decreased and was lower during medical air when compared with exercise performed during oxygen exposure (Figure 3).

## Discussion

This study reports, for the first time, the impact of hyperoxia on both APL and hemodynamic status in healthy volunteers at rest and during exercise. The main result of the study was that hyperoxia decreased APL in men, both at rest and during exercise. Our results and previous studies support the fact that APL is generated in an oxygen-dependent manner (Schrader et al., 1990). Indeed, it has been reported that hypoxia induced an increase in APL (Eltzschig et al., 2006; Joulia et al., 2013) whereas hyperoxia lead to a decrease in APL (Bruzzese et al., 2015; Fromonot et al., 2016).

The impact of adenosine on the hemodynamic status is well recognized and could contribute to the cardio-vascular changes observed in our healthy volunteers. In agreement with previous work, a decrease in CO secondary to a slowing of the heart rate was observed during hyperoxia (Waring et al., 2003; Gole et al., 2011). Furthermore, a significant increase in SVR was reported in healthy volunteers at rest during hyperoxia. The increase in oxygen partial pressure and the production of reactive oxygen species can be implicated in arterial vasoconstriction through an alteration in endothelial function or a direct effect on vascular smooth muscle (Welsh et al., 1998; Pasgaard et al., 2007). It has been shown that endothelial function was impaired after SCUBA diving, involving exposure to elevated partial pressures of oxygen at depth (Lambrechts et al., 2013). Pre-ingestion of nutritional antioxidants such as red orange extract (Balestra et al., 2016) or dark chocolate (Theunissen et al., 2015) prevented endothelial dysfunction. This improvement has been attributed, by the authors, to an increase in activity and expression of endothelial NO synthase. The decrease in APL might contribute to the vasomotor action of hyperoxia. Indeed, APL is recognized as having an impact on vasomotor tone, an increase in APL leading to vasodilation (Duza and Sarelius, 2003; Mortensen et al., 2009). The vasodilatory properties of adenosine are mediated by several physiological factors including an increase in NO production by endothelial cells (Li et al., 1995).

In our study, the slowing of the heart rate under hyperoxia might be secondary to the baroreflex stimulation induced by vasoconstriction (Waring et al., 2003; Demchenko et al., 2013). During exercise breathing medical air, the hemodynamic changes were common, including an increase in CO associated with a decrease in SVR. Furthermore, a significant increase in APL was observed, which could participate in the hyperemia induced by exercise through the action on arterial function (Hellsten et al., 2012).

In the session breathing pure oxygen, a significant increase in APL was recorded during exercise when compared with the resting condition. Nevertheless, APL remained lower than during exercise performed with medical air exposure. APL was comparable to the baseline measurements (in ambient air or during medical air exposure). Hemodynamic status was also significantly different between the two sessions. Hyperoxia-induced vasoconstriction could explain the increase in both systolic blood pressure and SVR. It could also explain the decrease in heart rate recorded in healthy volunteers during oxygen exposure when compared with medical air exposure, through baroreflex stimulation. Consequently, our findings supported previous observations that breathing pure oxygen induced vasoconstriction and attenuated hyperemia secondary to dynamic exercise (Welch et al., 1977; González-Alonso et al., 2002).

Nevertheless, there was no difference between the increases in APL recorded during exercise between the two sessions. In our study, exercise bouts were performed at low intensity (40% of VO_2_ peak), under the ventilatory threshold. Such exercises are recognized not inducing cellular hypoxia. Previous studies have attributed hyperemia to the release of oxygen-dependent factors secondary to the cellular hypoxia induced by anaerobic exercise (Marshall, 2007). Our study did not support this single mechanism since, although the exercises were performed in aerobic conditions, an increase in APL associated with a decrease in SVR was observed. Furthermore, the magnitude of the changes in these two parameters was reduced but not eliminated during exercise with oxygen exposure. In these circumstances, the increase in intravascular purines should not be secondary to hypoxia but to mechanical stressors induced by exercise. Indeed, it has been reported that shear stress and mechanical compression could induce a release of ATP from endothelial cells, erythrocytes and skeletal muscle cells (Sprague et al., 1996; Buvinic et al., 2009; Mortensen et al., 2011; Crecelius et al., 2013). The dephosphorylation of ATP by ectoenzymes CD39 and CD73 could explain the increase in APL (Borea et al., 2018).

### Study Limits

It is recognized that hyperoxic exposure during exercise impacted on both gas exchanges and minute ventilation (Ulrich et al., 2017). Cardiovascular function can be modified through the heart-lung interactions. Consequently, it would be interesting to perform a supplementary study on this topic.

## Conclusion

In our study, hyperoxia decreased APL in resting healthy volunteers but did not eliminate the increase in APL and the decrease in SVR during low intensity exercise. Further studies are needed to improve knowledge about the factors regulating the increase in APL during exercise according to the modalities (endurance or resistance, intensity) and its contribution to hyperemia.

## Data Availability Statement

The datasets generated for this study are available on request to the corresponding author.

## Ethics Statement

The studies involving human participants were reviewed and approved by Aix Marseille University, CPP-1, ID RCB: 2008-AOO171-54. The patients/participants provided their written informed consent to participate in this study.

## Author Contributions

AB, RG, and OG conceived and designed the study. NV and SR assisted with the technical aspects of the protocol, recruited all the participants, and involved in the acquisition of the data. MM and RG performed the biological study. AB and GC analyzed the data and performed the statistical analysis. AB, GC, and OG have drafted the manuscript while NV and RG revised it critically for important intellectual content. All authors have given final approval of the version to be published.

## Conflict of Interest

The authors declare that the research was conducted in the absence of any commercial or financial relationships that could be construed as a potential conflict of interest.
